# Association between health literacy and metabolic syndrome or healthy lifestyle characteristics among community-dwelling Japanese people

**DOI:** 10.1186/s13098-016-0142-8

**Published:** 2016-03-24

**Authors:** Hirohide Yokokawa, Hiroshi Fukuda, Motoyuki Yuasa, Hironobu Sanada, Teruhiko Hisaoka, Toshio Naito

**Affiliations:** Department of General Medicine, Juntendo University School of Medicine, Hongo 2-1-1, Bunkyo-ku, Tokyo, 113-8421 Japan; Department of Public Health, Juntendo University School of Medicine, Hongo 2-1-1, Bunkyo-ku, Tokyo, 113-8421 Japan; Division of Health Science Research, Fukushima Welfare Federation of Agricultural Cooperatives, Sakamizu 50, Aizubange Town, Fukushima, 969-6593 Japan; Department of Tumor and Host Bioscience, Fukushima Medical University School of Medicine, Hikarigaoka 1, Fukushima, 960-1295 Japan

**Keywords:** Epidemiology, Health Promotion, Health literacy, Metabolic syndrome, Lifestyle

## Abstract

**Background:**

Few studies have assessed the association between health literacy (HL) and healthy lifestyle characteristics among Japanese people, and reports on the association between HL and prevalence of metabolic syndrome are also scarce.

**Methods:**

The present cross-sectional study included 1817 (781 men and 1036 women) Japanese individuals who participated in a medical health checkup at Bange Kosei General Hospital and Takada Kosei Hospital in Fukushima, Japan, from April 2013–2014. Information regarding HL and healthy lifestyle characteristics listed in Breslow’s seven health practices was collected by self-administered questionnaire.

**Results:**

In multivariate logistic analysis, higher HL (≥14) was positively associated with healthy lifestyle characteristics [odds ratio (OR) = 2.08, 95 % confidence interval (CI) = 1.33–3.23] and inversely associated with prevalence of metabolic syndrome (OR = 0.67, 95 % CI = 0.48–0.95) among men. Among HL items, the ability to make decisions based on health-related information was significantly associated with healthy lifestyle characteristics (OR = 2.04, 95 % CI = 1.34–3.10 for men, OR = 1.38, 95 % CI = 1.30–1.85 for women) and inversely associated with prevalence of metabolic syndrome (OR = 0.62, 95 % CI = 0.44–0.88 for men, OR = 0.68, 95 % CI = 0.49–0.95 for women) in both sexes.

**Conclusions:**

We found positive associations between HL and healthy lifestyle characteristics and an inverse association with prevalence of metabolic syndrome among men. Our findings also suggest that men and women are likely to engage in health-promoting behaviors and make decisions based on health-related information. These findings highlight the importance of comprehensive assessments, including HL, for health promotion in the community.

## Background

In recent decades, both developed and developing countries have faced a dramatic increase in the number of adults suffering from non-communicable diseases (NCDs) such as diabetes, cardiovascular disease (CVD), and chronic kidney disease (CKD) [1]. Metabolic syndrome, in particular, has received considerable attention [2–4]. Metabolic syndrome is a concurrence of disturbed glucose and insulin metabolism, abdominal fat distribution, mild dyslipidemia, and hypertension [4], and is an important clinical condition given its association with the subsequent development of type 2 diabetes mellitus and cardiovascular disease [2–4]. The prevalence of metabolic syndrome and suspected individuals is estimated to be 20.1 million in Japan among those aged ≥40 years in 2008 [5]. Metabolic syndrome is known to increase the risk of mortality from coronary heart disease (CHD) and cardiovascular disease (CVD), and is also a risk factor for cognitive impairment [6, 7]. Therefore, modification of lifestyle characteristics at the individual level has been emphasized in the prevention of this syndrome.

Although the growing human and economic costs of metabolic syndrome have been recognized, and action plans to prevent it have been developed, the impact on health outcomes has been limited [5]. Unhealthy lifestyles likely contribute to the development of metabolic syndrome, and the importance of lifestyle modification has been emphasized through advanced communication tools [8].

Health information is important for people to understand and engage in the management of their own health. With an increasing amount of health information available through media reports and the internet, many health information resources can easily be distributed to the general population [9–11]. However, adequate use of these resources depends on an individual’s skill in finding and applying the information [12]. These skills have been conceptualized as “health literacy” (HL). Based on the World Health Organization (WHO) definition, a model of HL has been proposed that includes three levels, and assumes both individual and population benefits at each level: functional, communicative, and critical literacy [13]. Individuals with adequate HL are considered likely to adopt healthy lifestyles [14], and a brief questionnaire was developed to easily assess HL [15]. However, only a few studies have estimated the association between HL and healthy lifestyle characteristics among Japanese people [15–17], and reports on the association between HL and prevalence of metabolic syndrome are especially limited.

This study aimed to examine sex-specific associations between HL and healthy lifestyle characteristics and prevalence of metabolic syndrome among a Japanese community-dwelling population using a concise, newly developed questionnaire.

## Methods

### Participants

The present cross-sectional study included 1825 Japanese individuals who participated in a medical health checkup at Bange Kosei General Hospital and Takada Kosei Hospital in Fukushima, Japan, from April 2013–2014. Of these, eight were excluded due to refusal to participate or missing data. Thus, a total of 1817 participated in this study.

### Variables

Body height and weight were measured in the standing position. Body mass index (BMI) was calculated based on body weight (kg) divided by height squared (m^2^). Both systolic (SBP) and diastolic blood pressure (DBP) were obtained as the mean of two measurements on the upper arm after the participant had been seated for at least 5 min. We also collected information for the following lipid-related items: total cholesterol (mg/dL; TC), high-density lipoprotein-cholesterol (mg/dL; HDL-C), and triglycerides (mg/dL; TG). Low-density lipoprotein-cholesterol (mg/dL; LDL-C) was estimated using the Friedwald equation ([TC]–[HDL-C]–[TG/5]) [18]. We measured fasting plasma glucose (FPG) concentrations by the hexokinase ultraviolet method using an automated analyzer after more than 9 h of fasting.

We diagnosed metabolic syndrome according to the National Cholesterol Education Program’s Adult Treatment Panel III report (ATP III) [19]. For waist circumference, we diagnosed abdominal obesity using Japan-specific criteria (male waist circumference ≥90 cm, female waist circumference ≥80 cm), in accordance with the International Diabetes Federation (IDF) Worldwide Definition of metabolic syndrome [4]. When 3 out of 5 of the following listed characteristics are present, a diagnosis of metabolic syndrome is made: (1) abdominal obesity (male waist circumference ≥90 cm, female waist circumference ≥80 cm), (2) raised TG level (≥150 mg/dL) or specific treatment for this lipid abnormality, (3) reduced HDL-C level (≤40 mg/dL in men, ≤50 mg/dL in women) or specific treatment for this lipid abnormality, (4) raised blood pressure (systolic BP ≥130 mmHg or diastolic BP ≥85 mmHg) or treatment for previously diagnosed hypertension, and (5) raised fasting plasma glucose (≥110 mg/dL) or previously diagnosed type 2 diabetes [19].

We interviewed participants regarding their medical histories for atherosclerotic complications (cardiovascular or cerebrovascular diseases) and lifestyle-related disorders (hypertension, dyslipidemia, or diabetes mellitus). We then asked participants to complete self-administrated questionnaires, which included HL and healthy lifestyle characteristics listed in Breslow’s seven health practices, such as alcohol consumption, smoking behavior, exercise frequency, obesity (BMI), sleep duration, breakfast, and snacks between meals [20].

To evaluate HL, a validated questionnaire that included three items for communicative HL (items 1–3) and two items for critical HL (items 4, 5) was used [15]. A previous study using the questionnaire reported Cronbach’s α of communicative and critical HL scales to be 0.77 and 0.65, respectively [15]. Participants could easily complete the questionnaire because it has only five items. These items asked whether the participant would be able to (1) collect health-related information from various sources, (2) extract the relevant information, (3) understand and communicate the obtained information, (4) consider the credibility of the information, and (5) make decisions based on the information, specifically in the context of health-related issues. The participant rated each item on a 5-point Likert scale ranging from 1 (strongly disagree) to 5 (strongly agree).

We defined healthy lifestyle characteristics as the following responses based on Breslow’s seven health practices [20, 21]: alcohol consumption (non-everyday drinker), smoking behavior (non-current smoker), exercise frequency (twice or more times per week), BMI (18.5–24.9), sleep duration (7–8 h), breakfast (every morning), and snacks between meals (no).

### Statistical analysis

Variables are presented as mean ± standard deviation (SD) for continuous variables or prevalence (%) for categorical variables. Total HL score was dichotomized based on the median score (low HL < 14, high HL ≥ 14). We used the t test for continuous variables and the Chi square test or Fisher’s exact test for comparisons of proportions between sexes. To estimate the potential of HL to promote a healthy lifestyle with 6–7 healthy characteristics, which are most likely to be present in the healthiest lifestyle [21], logistic regression analysis was performed using the following models. Model 1 was adjusted for age (years), total HL score (≥14 vs. <14), and atherosclerotic complications (cardiovascular and cerebrovascular diseases). Model 2 was adjusted for age (years), five HL item scores (≥4 vs. <4), and atherosclerotic complications (cardiovascular and cerebrovascular diseases).

Logistic regression analysis was then performed to estimate associations between metabolic syndrome and total HL score (≥14 vs. <14) or five HL item scores (≥4 vs. <4) for both sexes. Model 1 was adjusted for age (years), total HL score (≥14 vs. ≤14), atherosclerotic complications (cardiovascular and cerebrovascular diseases), alcohol consumption (non-everyday drinker), and smoking behavior (non-current smoker). Model 2 was adjusted for age (years), five HL item scores (≥4 vs. ≤4), atherosclerotic complications (cardiovascular and cerebrovascular diseases), alcohol consumption (non-everyday drinker), and smoking behavior (non-current smoker).

All statistical analyses were performed using the Statistical Package for Social Sciences version 22 (IBM SPSS Inc., Chicago, IL, USA). *P* < 0.05 was considered statistically significant.

### Ethical considerations

This survey was conducted according to the Ethical Guidelines for Epidemiological Studies established by the Japanese government [22], and the Ethics Committee of Juntendo University approved the research protocol (No. 833). We obtained informed consent from all participants.

## Results

Table 1 summarizes the basic characteristics of male participants stratified by HL level. Among men, mean age was 51.2 years in the lower HL group, and 51.0 years in the higher HL group. TG and FPG were significantly lower in the higher HL group compared to the lower HL group. Although proportions of those with metabolic syndrome, waist circumference (≥90 cm), and raised fasting plasma glucose (≥110 mg/dL) did not significantly differ between higher and lower HL groups, the differences were nearly significant (*P* = 0.06, 0.08, and 0.05, respectively). As for healthy lifestyle characteristics, proportions of smoking behavior (non-smoker), BMI (18.5–24.9), and exercise frequency (twice or more per week) were also significantly higher in the higher HL group compared to the lower HL group. In addition, the proportion of participants with 6–7 total healthy lifestyle items was significantly higher in the higher HL group. Among women, mean age was 49.2 years in lower and higher HL groups. There were no significant differences between higher and lower HL groups in any of the basic characteristics (Table 2). The prevalence of metabolic syndrome in all participants, men, and women were 15.6, 20.9, and 11.6 %, respectively.

**Table 1:** Health literacy specific characteristics among men (N = 781)

	Mean (±SD) or N (%)		*P* ^a^
	Low health literacy (≤13) (N = 350)	High health literacy (≥14) (N = 431)	
Age (years)	51.2 (9.9)	51.0 (9.9)	
Anthropometric measurements			
Height (cm)	170.0 (6.5)	170.2 (6.4)	
Body weight (kg)	68.6 (12.1)	68.8 (11.1)	
Body mass index (BMI)	23.7 (3.8)	23.7 (3.4)	
Waist circumference (cm)	85.0 (9.5)	84.6 (8.8)	
Atherosclerotic complications			
Cardiovascular disease	13 (3.7)	18 (4.2)	
Cerebrovascular disease	3 (0.9)	8 (1.9)	
Hypertension-related factors			
Systolic blood pressure (mmHg)	133.5 (19.4)	135.0 (17.7)	
Diastolic blood pressure (mmHg)	81.1 (12.4)	81.7 (12.1)	
Antihypertensive drug use (yes)	72 (20.6)	102 (23.7)	
Lipid-related items			
High-density lipoprotein cholesterol (mg/dL)	57.1 (15.2)	58.9 (15.5)	
Low-density lipoprotein cholesterol (mg/dL)	130.1 (32.7)	128.2 (33.0)	
Triglycerides (mg/dL)	168.1 (132.5)	149.4 (106.4)	*
Antidyslipidemic drug use (yes)	29 (8.3)	41 (9.5)	
Diabetes-related items			
Fasting plasma glucose (mg/dL)	105.1 (27.1)	101.3 (18.4)	*
Antidiabetic drug use (yes)	12 (3.4)	17 (3.9)	
Metabolic syndrome (yes)	80 (24.0)	75 (18.3)	^b^
Waist circumference (≥90 cm)	107 (32.0)	107 (26.2)	^b^
Raised triglyceride (≥150 mg/dL)	132 (37.7)	149 (34.6)	
Reduced high density lipoprotein cholesterol (≤40 mg/dL)	25 (7.1)	26 (6.0)	
Raised blood pressure (systolic ≥130 mmHg or diastolic ≥85 mmHg)	228 (65.3)	301 (69.8)	
Raised fasting plasma glucose (≥110 mg/dL)	83 (23.7)	78 (18.1)	^b^
Healthy lifestyle characteristics			
Alcohol consumption (non-everyday drinker)	161 (46.0)	223 (51.7)	
Smoking behavior (non-current smoker)	174 (49.7)	262 (60.8)	**
Exercise frequency (2 times or more per week)	48 (13.7)	96 (22.3)	**
Body mass index (18.5–24.9)	216 (61.7)	293 (68.0)	*
Sleep hours (6–9)	220 (62.9)	290 (67.3)	
Breakfast (every morning)	277 (79.1)	355 (82.4)	
Snack between meals (no)	287 (82.0)	371 (86.1)	
Total number of healthy lifestyle items	4.0 (1.2)	4.4 (1.2)	**
Proportion of participants with 6 or 7 total number of healthy lifestyle items	34 (9.7)	76 (17.6)	**
Health literacy			
Seeking information from various sources	3.3 (1.0)	4.1 (0.6)	**
Extracting relevant information	3.0 (0.9)	4.0 (0.5)	**
Understanding and communicating the information	2.7 (0.7)	3.9 (0.5)	**
Considering the credibility of the information	2.7 (0.7)	3.8 (0.5)	**
Making decisions based on the information	2.8 (0.8)	3.7 (0.6)	**
Total scale score	11.1 (2.1)	15.5 (1.3)	**

^a^** *P* < 0.01, * *P* < 0.05

^b^Borderline significance 0.05 < *P* < 0.10

**Table 2:** Health literacy specific characteristics among women (N = 1036)

	Mean (± SD) or N (%)		*P* ^a^
	Low health literacy (<14) (N = 437)	High health literacy (≥14) (N = 599)	
Age (years)	49.2 (9.7)	49.2 (9.5)	
Anthropometric measurements			
Height (cm)	157.4 (6.3)	157.4 (5.8)	
Body weight (kg)	55.5 (9.8)	55.1 (9.3)	
Body mass index (BMI)	22.4 (3.9)	22.3 (3.7)	
Waist circumference (cm)	80.2 (9.8)	80.1 (9.3)	
Atherosclerotic complications			
Cardiovascular disease (yes)	3 (0.7)	9 (1.5)	
Cerebrovascular disease (yes)	5 (1.1)	4 (0.7)	
Hypertension-related factors			
Systolic blood pressure (mmHg)	124.7 (18.4)	124.9 (18.8)	
Diastolic blood pressure (mmHg)	76.1 (11.1)	76.0 (11.4)	
Antihypertensive drug use (yes)	58 (13.3)	69 (11.5)	
Lipid-related items			
High-density lipoprotein cholesterol (mg/dL)	71.3 (17.5)	70.9 (16.2)	
Low-density lipoprotein cholesterol (mg/dL)	122.4 (32.0)	123.7 (31.4)	
Triglycerides (mg/dL)	92.7 (53.6)	92.4 (57.3)	
Antidyslipidemic drug use (yes)	42 (9.6)	52 (8.7)	
Diabetes-related items			
Fasting plasma glucose (mg/dL)	94.3 (17.3)	93.8 (17.0)	
Antidiabetic drug use (yes)	8 (1.8)	10 (1.7)	
Metabolic syndrome (yes)	53 (12.5)	64 (11.0)	
Waist circumference (≥80 cm)	216 (50.9)	285 (49.0)	
Raised triglyceride (≥150 mg/dL)	50 (11.4)	60 (10.0)	
Reduced high density lipoprotein cholesterol (≤40 mg/dL)	29 (6.6)	43 (7.2)	
Raised blood pressure (systolic ≥130 mmHg or diastolic ≥85 mmHg)	204 (46.7)	252 (42.1)	
Raised fasting plasma glucose (≥110 mg/dL)	33 (7.6)	34 (5.7)	
Healthy lifestyle characteristics			
Alcohol consumption (non-everyday drinker)	361 (82.6)	509 (85.0)	
Smoking behavior (non-current smoker)	371 (84.9)	516 (86.1)	
Exercise frequency (2 times or more per week)	32 (7.3)	58 (9.7)	
Body mass index (18.5–24.9)	307 (70.3)	406 (67.8)	
Sleep hours (6–9)	234 (53.5)	323 (53.9)	
Breakfast (every morning)	365 (83.5)	512 (85.5)	
Snack between meals (no)	329 (75.3)	446 (74.5)	
Total number of healthy lifestyle items	4.6 (1.2)	4.6 (1.2)	
Proportion of participants with 6 or 7 total number of healthy lifestyle items	97 (22.2)	148 (24.7)	
Health literacy			
Seeking information from various sources	3.5 (0.9)	4.2 (0.6)	**
Extracting relevant information	3.1 (0.9)	4.1 (0.5)	**
Understanding and communicating the information	2.7 (0.7)	3.9 (0.5)	**
Considering the credibility of the information	2.6 (0.7)	3.7 (0.6)	**
Making decisions based on the information	2.8 (0.7)	3.8 (0.6)	**
Total scale score	11.2 (2.0)	15.5 (1.3)	**

^a^ ** *P* < 0.01, * *P* < 0.05

Table 3 show multivariate regression analyses for associations between HL and 6–7 healthy lifestyle characteristics. Among men, a higher HL (total scale score ≥14) was significantly associated with having 6–7 healthy lifestyle characteristics in Model 1 [odds ratio (OR) = 2.08, 95 % confidence interval (CI) = 1.33–3.23)]. In addition, “Understanding and communicating the information” and “Making decisions based on the (health-related) information” were significantly associated with having 6–7 healthy lifestyle characteristics in Model 2 [(OR = 1.63, 95 % CI = 1.08–2.47), (OR = 2.04, 95 % CI = 1.34–3.10)] (Table 3). Among women, no association was observed between HL and healthy lifestyle characteristics (Table 3).

**Table 3:** Logistic regression analysis of health literacy for men and women with 6–7 healthy lifestyle characteristics

	Univariate analysis			Multivariate analysis					
				Model 1^c^			Model 2^d^		
	OR^a^	95 % CI^b^	*P* ^e^	OR^a^	95 % CI^b^	*P* ^e^	OR^a^	95 % CI^b^	P^e^
For men (N = 781)									
Health literacy									
Seeking information from various sources (≥4 vs. <4)	1.26	0.78–2.02		–	–		1.44	0.89–2.34	
Extracting relevant information (≥4 vs. <4)	1.31	0.85–2.01		–	–		1.50	0.97–2.32	
Understanding and communicating the information (≥4 vs. <4)	1.59	1.06–2.39	**	–	–		1.63	1.08–2.47	*
Considering the credibility of the information (≥4 vs. <4)	1.49	0.99–2.23		–	–		1.39	0.92–2.10	
Making decisions based on the information (≥4 vs. <4)	2.16	1.42–3.27	**	–	–		2.04	1.34–3.10	**
Total score (≥14 vs. <14)	1.99	1.29–3.06	**	2.08	1.33–3.23	**	–	–	
For women (N = 1036)									
Health literacy									
Seeking information from various sources (≥4 vs. <4)	0.87	0.61–1.23		–	–		1.05	0.73–1.51	
Extracting relevant information (≥4 vs. <4)	0.81	0.60–1.09		–	–		0.87	0.64–1.18	
Understanding and communicating the information (≥4 vs. <4)	1.07	0.80–1.42		–	–		0.98	0.73–1.31	
Considering the credibility of the information (≥4 vs. <4)	1.56	1.17–2.01	**	–	–		1.43	1.07–1.92	*
Making decisions based on the information (≥4 vs. <4)	1.54	1.15–2.05	**	–	–		1.38	1.30–1.85	**
Total score (≥14 vs. <14)	1.15	0.86–1.54		1.17	0.86–1.58		–	–	

^a^ Odds ratio

^b^ 95 % confidence interval

^c^ Model 1 was adjusted for age (years), total health literacy score (≥14 vs. <14), and atherosclerotic complications (cardiovascular and cerebrovascular diseases)

^d^ Model 2 was adjusted for age (years), five health literacy items (≥4 vs. <4), and atherosclerotic complications (cardiovascular and cerebrovascular diseases)

^e^ ** *P* < 0.01, * *P* < 0.05

As for metabolic syndrome, a higher HL (total scale score ≥14) was significantly associated with a lower prevalence of metabolic syndrome in Model 1 (OR = 0.67, 95 % CI = 0.48–0.95) among men (Table 4). “Making decisions based on the (health-related) information” was significantly associated with a lower prevalence of metabolic syndrome in Model 2 (OR = 0.62, 95 % CI = 0.44–0.88 for men, OR = 0.68, 95 % CI = 0.49–0.95 for women) in both sexes (Table 4).

**Table 4:** Logistic regression analysis of health literacy for men and women with metabolic syndrome

	Univariate analysis			Multivariate analysis					
				Model 1^c^			Model 2^d^		
	OR^a^	95 % CI^b^	*P* ^e^	OR^a^	95 % CI^b^	*P* ^e^	OR^a^	95 % CI^b^	P^e^
For men (N = 742)									
Health literacy									
Seeking information from various sources (≥4 vs. <4)	0.83	0.56–1.21		–	–		0.75	0.52–1.09	
Extracting relevant information (≥4 vs. <4)	0.82	0.58–1.18		–	–		0.80	0.55–1.11	
Understanding and communicating the information (≥4 vs. <4)	0.80	0.57–1.14		–	–		0.80	0.57–1.13	
Considering the credibility of the information (≥4 vs. <4)	0.82	0.58–1.16		–	–		0.80	0.55–1.10	
Making decisions based on the information (≥4 vs. <4)	0.64	0.45–0.91	*	–	–		0.62	0.44–0.88	**
Total score (≥14 vs. <14)	0.69	0.49–0.97	*	0.67	0.48–0.95	**	–	–	
For women (N = 1006)									
Health literacy									
Seeking information from various sources (≥4 vs. <4)	0.62	0.43–0.89	**	–	–		0.81	0.55–1.19	
Extracting relevant information (≥4 vs. <4)	0.67	0.48–0.93	*	–	–		0.81	0.57–1.13	
Understanding and communicating the information (≥4 vs. <4)	0.88	0.64–1.21		–	–		0.85	0.61–1.18	
Considering the credibility of the information (≥4 vs. <4)	0.97	0.70–1.33		–	–		0.89	0.64–1.24	
Making decisions based on the information (≥4 vs. <4)	0.80	0.59–1.10		–	–		0.68	0.49–0.95	*
Total score (≥14 vs. <14)	0.82	0.60–1.13		0.80	0.57–1.12		–	–	

^a^Odds ratio

^b^95 % confidence interval

^c^Model 1 was adjusted for age (years), total health literacy score (≥14 vs. ≤14), atherosclerotic complications (cardiovascular and cerebrovascular diseases), alcohol consumption (non-everyday drinker), and smoking behavior (non-current smoker)

^d^Model 2 was adjusted for age (years), five health literacy items (≥4 vs. ≤4), atherosclerotic complications (cardiovascular and cerebrovascular diseases), alcohol consumption (non-everyday drinker), and smoking behavior (non-current smoker)

^e^ ** P < 0.01, * P < 0.05

## Discussion

Our analysis of cross-sectional data showed that higher HL was significantly associated with healthy lifestyle characteristics, specifically non-smoker status and high exercise frequency, among men. Moreover, the proportion of metabolic syndrome was lower and the proportion of ideal BMI (18.5–24.9) was higher with higher HL compared with lower HL among men. Multivariate analyses revealed that higher HL (≥14) was significantly associated with having 6–7 healthy lifestyle characteristics, and was also inversely associated with metabolic syndrome among men, whereas no significant association was found among women. The ability to make decisions based on health-related information was positively associated with a healthy lifestyle and inversely associated with metabolic syndrome in both sexes. Similar to our results, median scores of communicative and critical HL in a previous study that used the same scales were both 4 [13]. Thus, our study population may be similar to the general Japanese population. To the best of our knowledge, this study is the first to examine associations between HL and healthy lifestyle characteristics and prevalence of metabolic syndrome among Japanese people. Our results highlight the importance of improving HL to prevent metabolic syndrome through a healthy lifestyle.

Only a few studies have examined the association between HL and obesity, between HL and metabolic syndrome, and between HL and BMI among adult individuals [20, 21]. A cross-sectional study, which was conducted in the United States using the Newest Vital Sign (NVS) to measure HL, reported that BMI predicted adequate HL among adults (OR = 1.040, 95 % CI = 1.016–1.065) [23]. In another cross-sectional survey, which examined the association between HL measured by NVS and BMI among 364 native Hawaiian and Pacific Islanders in the United States, low NVS scores were associated with increased BMI (*r* = −0.12, P = 0.027) [24]. Associations between HL and child and adolescent obesity have also been reported [25, 26]. In one cross-sectional study that tested the association between child and parental HL (using NVS) and their obesity, children’s obesity decreased with higher parental NVS (OR = 0.75, 95 % CI = 0.59–1.00), and increased with parental obesity (OR = 2.53, 95 % CI = 1.08–5.94) [25]. In a survey that examined the association between child HL using the Short Test of Functional Health Literacy in Adults (STOFHLA) and BMI among 171 overweight children in the United States, child STOFHLA correlated negatively with BMI Z score (*r* = −0.37, P < 0.01) [26]. In this study, we also found that the proportion of ideal BMI (18.5–24.9) was higher in the higher HL group than in the lower HL group among men. This suggests that a higher HL may contribute to maintaining an ideal body weight. Thus, estimation of HL level may be useful and even necessary for lifestyle modification strategies.

We also found an association between HL level and prevalence of metabolic syndrome among men. Metabolic syndrome represents clusters of cardiovascular risk factors, assuming that cardiovascular risk is amplified more than is expected from the effect of single risk factors [2–4, 27]. The risk of cardiovascular events and death was found to be markedly greater for individuals with metabolic syndrome compared to those without, regardless of the definition of metabolic syndrome [27]. Thus, preventing metabolic syndrome will be important for the prevention of cardiovascular disease. To date, only a few studies have assessed the association between metabolic syndrome and HL level, and correlations with chronic disease-related HL [28]. Additional studies will be needed to further explore these associations and correlations.

We also found an association between HL level and healthy lifestyle characteristics among men. In a previous Japanese cross-sectional study, which examined the relationship between HL assessed by the 14-item Health Literacy Scale (HLS-14) and health information access, health behavior, and health status, HL was reported to be associated with health information access and health behavior [29]. The authors emphasized the importance of raising HL levels of the target population for health promotion intervention [29]. In another study, which examined the mechanisms linking HL to physical activity and self-reported health among 330 patients with hypertension, significant paths were found from HL to knowledge (r = 0.22, P < 0.001), knowledge to self-efficacy (r = 0.13, P < 0.01), self-efficacy to physical activity (r = 0.17, P < 0.01), and physical activity to health status (r = 0.17, P < 0.01) [30]. Thus, a higher HL may be related with healthier lifestyle characteristics, which in turn can contribute to a lower prevalence of metabolic syndrome among men.

Among the five assessed items in this study, “Making decisions based on the (health-related) information” was significantly associated with metabolic syndrome and healthy lifestyle characteristics in men. The National Panel of people with Chronic illness or Disability (NPCD) in the Netherlands reported that communicative and critical HL are related to some aspects of self-management [31]. Consistent with this, a cross-sectional study in Japanese primary care showed that communicative and critical HL were positively associated with an understanding of diabetes care (β = 0.558, 0.451, P < 0.001) and self-efficacy (β = 0.365, 0.369, P < 0.001), respectively [32]. Thus, critical HL may be related with metabolic syndrome as well as healthy lifestyle characteristics through self-management ability.

We did not find an association between HL level and metabolic syndrome among women in the present study. One potential explanation is that women are likely to have healthier lifestyle characteristics, especially in terms of alcohol consumption and smoking, compared to men. Moreover, the prevalence of metabolic syndrome is lower in women than in men. These differences may explain the lack of association. Further studies will be needed to confirm this.

This study has several limitations. First, there may have been selection bias, as study participants were recruited from those who participated in a health checkup program at two Japanese hospitals. Thus, these participants may have had a higher health awareness compared to non-participants. Large-scale studies that include several regions in Japan and also include individuals who have not received health checkups will be needed in the future. Second, HL and lifestyle characteristics were measured based on self-reported questionnaires. It is possible that these participants may have reported better HL and healthier lifestyles than is actually true, thereby resulting in an over-estimation of HL and health characteristics. Third, given the cross-sectional design, we could not determine whether there was a causal relationship between HL and healthy lifestyle characteristics. A longitudinal study will be needed to address this issue. Fourth, while the information collected from participants contained a comprehensive set of clinical variables, it cannot be denied that some important factors, especially educational status, were not measured.

## Conclusions

Our study revealed a positive association between HL and healthy lifestyle characteristics and an inverse association with prevalence of metabolic syndrome among men. Our findings also suggest that men and women are likely to engage in health-promoting behaviors and make decisions based on health-related information. These findings highlight the importance of comprehensive assessments, including HL, for health promotion in the community, and the need to improve HL in order to prevent metabolic syndrome through a healthy lifestyle.
